# What Really Matters for Loneliness Among Left-Behind Children in Rural China: A Meta-Analytic Review

**DOI:** 10.3389/fpsyg.2019.00774

**Published:** 2019-04-18

**Authors:** Xiaoyun Chai, Hongfei Du, Xiaoyan Li, Shaobing Su, Danhua Lin

**Affiliations:** ^1^Institute of Developmental Psychology, Beijing Normal University, Beijing, China; ^2^Department of Psychology, Social and Health Psychology Research Center, Guangzhou University, Guangzhou, China; ^3^Eliot-Pearson Department of Child Study and Human Development, Institute for Applied Research in Youth Development, Tufts University, Medford, MA, United States

**Keywords:** loneliness, left-behind children, predictors, meta-analysis, systematic review

## Abstract

In rural China, left-behind children are likely to suffer chronic loneliness. Research has identified a variety of factors that may be associated with loneliness among these children. A meta-analysis is needed to address the empirical inconsistencies and examine the strength of relations between different factors and loneliness. The current meta-analysis included 51 studies on predictors of loneliness published from 2008 to 2017. Results showed that one individual factor (social anxiety) is a key risk factor for loneliness, whereas eight individual (older age, self-esteem, resilience, extroversion) and contextual factors (family functioning, parent–child relationship, peer relationship, social support) serve as protective factors in predicting loneliness. In addition, boys were more likely to feel lonely than girls. Findings and implications of this study were discussed.

In some developing countries (e.g., China, Philippines, Mexico, Sri Lanka), millions of parents leave their children and migrate to other regions or countries for jobs (Wen and Lin, 2012; Givaudan and Pick, 2013; Siriwardhana et al., 2015). Leaving children behind has become a widespread phenomenon (Duan and Zhou, 2005; Dillon and Walsh, 2012) impacting children's development in both the short and long term (Lu and Treiman, 2011; Wen et al., 2015; Su et al., 2017). In China, *left-behind children* refer to those children under 18 years old who have been left-behind in their rural hometown when one or both parents migrate elsewhere to work (Duan and Zhou, 2005; Su et al., 2013). By the end of 2010, it was estimated that there were more than 61 million left-behind children, accounting for 37.70% of rural children and 21.88% of the child population in China (All China Women's Federation., 2013). Of these, 32.67% were in the care of their grandparents, 3.3% were cared for by other relatives, and 4% had no guardian at all (All China Women's Federation., 2013). Whereas parental migration brings economic benefits to left-behind children, it has deleterious impacts on the development of these children (Luo et al., 2009; Antón, 2010; Givaudan and Pick, 2013; Nguyen, 2016). Previous meta-analyses focusing on left-behind children in China suggested that parental migration had negative impacts on children's mental health, psychological well-being, and academic achievements (Wang and Mesman, 2015; Zhao and Yu, 2016), which is consistent with research on left-behind children in other countries (e.g., Philippines, Sri Lanka, Ecuador, Mexico) (Valtolina and Colombo, 2012).

Loneliness, which is conceptualized as an aversive state of discrepancy between desired and experienced social relationships (Peplau and Perlman, 1982), is identified as a typical developmental problem that left-children are likely to experience (Shen et al., 2015). The theory of loneliness and social connection posits that weak family connections are associated with emotional and social loneliness (Weiss, 1973; Cacioppo et al., 2015). According to this perspective, left-behind children who have experienced prolonged physical separation with their parent(s), are more vulnerable to loneliness. Indeed, a survey conducted in six provinces in China found that ~25% of left-behind children reported high levels of loneliness (Yin, 2014). In a cross-sectional study, left-behind children were 2.5 times more likely to suffer from loneliness, compared to children of non-migrant families (Jia and Tian, 2010); furthermore, compelling evidence from a meta-analysis by Chen et al. (2017) showed that left-behind children had a higher level of loneliness than their counterparts (*d* = 0.29).

A chronic and painful state of loneliness is harmful to mental and physical health (Heinrich and Gullone, 2006; Qualter et al., 2015; Lempinen et al., 2018); in fact, it may elevate the risk for depression (Cacioppo et al., 2015), mortality (Holt-Lunstad et al., 2015), social withdrawal, and suicidality (Schinka et al., 2013), even damaging the immune, cardiovascular, and nervous systems (Cacioppo et al., 2015; LeRoy et al., 2017). As for left-behind children, a sense of yearning for their parents and chronically high loneliness result in a constellation of mental problems, including conduct problems (Yu, 2017) and suicide attempts (Chang et al., 2017).

Recognizing the detrimental impact of loneliness in the development of left-behind children, an increasing number of studies have focused on individual and contextual factors that are related to loneliness, such as gender (Fan et al., 2016), age (Yue et al., 2014), self-esteem (Song et al., 2017), and family functioning (Zhao, 2013). However, a few gaps exist in the literature. First, mixed findings have been found in terms of predictors (e.g., gender). Second, most research showed a lack of theoretical framework about the pathways between these factors and loneliness. Third, based on these studies, it is difficult to tell what factors are more important for left-behind children. To address these gaps, a theory-based meta-analytic approach can be used to review the literature and to examine the influence of multiple factors on loneliness. Such an approach may contribute to interventions and policies that aim to reduce the risk of loneliness among left-behind children in China. Although a previous meta-analysis has identified some individual and contextual variables (e.g., age, self-esteem, social support) that are associated with loneliness in adolescence (Mahon et al., 2006), it is unclear whether these findings could account for loneliness among left-behind children.

Accordingly, we conducted a meta-analysis on individual and contextual factors associated with loneliness among left-behind children in China. The present research is grounded in the ecological systems framework, which emphasizes the connections between individual and environmental systems in understanding human development (Bronfenbrenner, 1979). According to the ideas of ecological systems framework, we should integrate multiple processes of individual functioning and multiple developmental contexts to better understand the risk or protective factors for loneliness among left-behind children. To be more specific, demographic, and intrapersonal psychological variables can be organized as individual level factors, and family-, school-, or community-related variables can be organized as contextual level factors. With respect to left-behind children, many individual and contextual factors have been found to be associated with loneliness (Shen et al., 2015). Next, we will give an overview of individual and contextual factors that have been identified to be associated with loneliness among left-behind children.

## Factors Associated with Loneliness in Left-Behind Children

Loneliness has been found to be associated with a variety of individual factors of left-behind children, including demographic characteristics (e.g., gender, age) (Liu et al., 2008; Zhao and Shen, 2011), intrapersonal psychological factors (e.g., self-esteem) (Fan et al., 2014), and emotion-related problems (e.g., social anxiety) (Liao et al., 2014). To be specific, age may alter children's vulnerability to loneliness. Existing studies suggested that older left-behind children may experience lower levels of loneliness (Zhao and Shen, 2011; Yue et al., 2014). Gender also has been regarded to play a role in the development of loneliness among left-behind children. Previous findings on the association between them were, however, mixed: some researchers found that boys report more loneliness that of girls (Xu, 2008; Sun et al., 2010; Fan et al., 2016), whereas others studies found no gender difference or opposite results (Liu et al., 2007; Qi and Jia, 2010). Besides, some intrapersonal psychological characteristics were found to correlate with lower levels of loneliness among left-behind children, including high self-esteem (Fan et al., 2014), resilience (Ai and Hu, 2016), psychological capital (Fan et al., 2017), positive appraisals of adversity (Zhao et al., 2013), positive coping styles (Liao et al., 2014), extroversion (Fan et al., 2014), and hope (Fan et al., 2016). Thus, these inherent factors may play important roles in preventing loneliness. In addition, experiencing social anxiety and feeling lonely are common and interrelated internalizing problems in child and adolescence (Jones et al., 1990). Existing studies have noted that social anxiety was positively associated with loneliness among left-behind children (Yuan et al., 2014; Ren et al., 2017). These findings may imply that social anxiety is a risk factor for experiencing loneliness or, vice versa, that feeling lonely aggravates children's social anxiety.

In addition, a growing concern has arisen that many factors within their ecological contexts (e.g., family, school) can have a substantial influence in loneliness among left-behind children (Shen et al., 2015). Family is one of the key contexts that may provide resources and or challenges that may influence children's perception of loneliness (Sharabi et al., 2012). Research has indicated that the levels of loneliness perceived by left-behind children are significantly related to how well their families function (Xie, 2008; He, 2010; Yue et al., 2014). Positive family functioning may protect left-behind children from the impacts of loneliness (Zhong et al., 2010) whereas a dysfunctional family atmosphere is associated with high levels of loneliness among these children (Fan et al., 2014). Moreover, high parental support and better parent-child relationships were also associated with low levels of loneliness among left-behind children (Liu et al., 2008; Zhao et al., 2015). In addition, parental migration status, which is often classified into two groups: both-parent migration and one-parent migration, may also be related to children's loneliness. Existing literature contains mixed findings on the role of parental migration status. Some studies found that children with both-parent migrating reported higher levels of loneliness than children with one-parent migrating (Duan, 2014a; Yue and Lu, 2015), whereas other studies found no difference in loneliness between these two groups (Qi and Jia, 2010; Su et al., 2013).

With regard to school context, the roles of teachers and peers are important in predicting these children's loneliness (Asher and Paquette, 2003; Galanaki, 2004). For example, multiple studies have shown that peer relationship is negatively associated with loneliness (Asher and Paquette, 2003; Chen et al., 2004; Vanhalst et al., 2014; Spithoven et al., 2017). Peer acceptance and high-quality friendships are associated with low levels of loneliness among left-behind children (Sun et al., 2010; Wang et al., 2011). In addition, some other school level factors, such as perceived support from peers and teachers (Liu et al., 2008; Zhang, 2011a), teacher-student relationships (Xu, 2008), and sense of belonging at school (Yang et al., 2016), are also related to loneliness among left-behind children.

## The Present Study

The aim of this study is to address the empirical inconsistencies and examine the strength of relations between different factors and loneliness among left-behind children using a meta-analytic approach based on the ecological systems framework. Although many studies have explored the influence of individual and contextual factors on loneliness among left-behind children, there is a need to review the literature and to evaluate the effects of key factors on loneliness based on numerous studies accumulated in this field. Understanding the influence of these factors in the experience of loneliness may inform intervention programs and social policies that focus on reducing the perception of loneliness among these children. In the present study, we used a meta-analytic approach to examine the influences of these factors.

## Methods

### Data Sources and Search Strategy

We conducted a systematic search of the literature in both Chinese and English using several electronic databases, including China National Knowledge Infrastructure (CNKI), PubMed, Web of Science, and PsycInfo. We also conducted a literature search by using Google Scholar and searched master's theses and doctoral dissertations through the China Dissertation Database and ProQuest Dissertations & Theses. The wide variety of key words we used included *left-behind child, left-behind adolescent, loneliness, predictor, protective, and risk factors* (A detailed description appears in Appendix A).

### Inclusion and Exclusion Criteria

We set the following criteria for articles to be included in this study: (a) the articles had to be empirical investigations of Chinese left-behind children's loneliness; (b) the study design had to be quantitative; (c) the articles had to be published or reported from 2000 to 2017 and available in Chinese or English. The year 2000 was chosen because the Chinese government and researchers initiated their focus on left-behind children issues at that time (Tan, 2011); and (d) the articles had to provide sufficient statistic information for the calculation or estimation of effect sizes (e.g., correlation, *t*-value, *F*-value, *p*-value). Articles were excluded on any of the following grounds: the studies (a) took the form of a review, a case study, a qualitative report, or a comment; and (b) reported only loneliness prevalence and did not examined individual or contextual predictors of loneliness.

### Coding of Studies and Quality Assessment

A coding protocol was designed to guide coding and information retrieval. The following information was extracted from each eligible study: author name, study design, sample size, gender, location, age range, age group (elementary school students: Grades 1–6 or age 6–12 years; junior high school students: Grades 7–9 or age 13–17 years), year of publication, publication type, measure of loneliness, and estimated effect size. The eligible studies were subjected to a methodological quality assessment by two coders (the first author and third author), using a 14-item instrument, a modified quality index based on prior literature (Downs and Black, 1998; Ferro and Speechley, 2009). One item (i.e., “Were the staff, places, and facilities where the patients were studied representative of the treatment the majority of patients receive?”) was deleted from Ferro and Speechley (2009) revised quality checklist because it was inappropriate in the context of left-behind children's loneliness. The quality checklist assessed four aspects of methodological quality: reporting (e.g., “Is the hypothesis/objective of the study clearly described?”), external validity (e.g., “Were the participants asked to participate in the study representative of the entire population from which they were recruited?”), internal validity (e.g., “Were the main outcome measures used valid and reliable?”), and power (“Did the study provide a sample size or power calculation to detect important effects where the probability value for a difference being due to chance is < 0.05?”). A detailed description of the modified quality index appears in Appendix B. Each item was scored 0 (no / unable to determine) or 1 (yes). The maximum score achievable was 14. Studies with higher scores indicated higher methodological quality. All eligible studies were reviewed by two coders to settle on the most appropriate coding. Differences in interpretation were resolved through discussion with a correspondence author to reach an agreement.

### Effect Size of Calculation

In the current review, we used Pearson's correlation coefficient *r* as the effect-size index for this meta-analysis. For studies that presented data as means and standard deviations, or inferential statistics, such as *t, F*, or *p-*values, results were converted to Pearson's correlation coefficient *r* using the *ES* calculator provided by Wilson (2001). For the effect size of a longitudinal study at several different time points, we chose the effect size of the time point with the largest sample size. Furthermore, according to the shifting unit of analysis approach (Cooper, 2010), the effect sizes of support from different sources (e.g., father, mother, peer, and teacher) (Liu et al., 2008) were combined into an effect size of social support; the effect sizes of father-child relationship and mother-child relationship (Zhang, 2011b) were combined into an effect size of parent-child relationship. We used Cohen's guidelines to interpret the effect size, where *r* of at least 0.10 = small, 0.30 = medium, and 0.50 = large (Cohen, 1992).

### Method of Meta-Analysis

A meta-analysis was conducted for each predictor where at least two independent studies reported a measure of effect size. Other predictors were excluded if only one study was available, including cognitive appraisals of struggles associated with being left-behind (Zhao and Shen, 2011), teacher-student relationship (Xu, 2008), sense of belonging at school (Yang et al., 2016), core self-evaluation (Zhao, 2015), dysfunctional family atmosphere (Fan et al., 2014), beliefs about adversity (Zhao et al., 2013), hope (Fan et al., 2016), psychological capital and stress (Fan et al., 2017), family abuse and neglect (Duan and Zhang, 2014), and coping styles (Liao et al., 2014). We performed this meta-analysis using comprehensive meta-analysis software (Borenstein et al., 2006). A separate meta-analysis was performed for each factor. In the meta-analysis we used random effects models. The assumption underlying fixed effects models is that one true effect size exists in all eligible studies, but random effects models allow that true effect could vary across studies (Borenstein et al., 2009). Random-effect meta-analyses were, therefore, generally more appropriate for review in this meta-analysis.

To examine the presence of heterogeneity, we computed the *Q* statistic (a measure of weighted squared deviations), *I*^2^ (the ratio of true heterogeneity to total observed variation), and τ^2^ (between-studies variance) (Borenstein et al., 2009). The following guidelines were used to interpret *I*^2^: low heterogeneity, *I*^2^ = 25%; moderate heterogeneity, *I*^2^ = 50%; high heterogeneity, *I*^2^ = 75% (Higgins et al., 2003).

Subgroup analysis was undertaken to explore whether potential moderator variables could account for significant variability among effect sizes. Four significant predictors of loneliness (gender, self-esteem, peer relationship, and social support) were tested for moderating effects. Other predictors (age, resilience, extroversion, social anxiety, family functioning, and parent–child relationship) were not considered in the subgroup analysis because of the small number of studies. Two potential moderator variables (i.e., age group and study quality) in each factor were tested. First, we tested age group difference in effect sizes because prior literature has shown that older age children experienced less loneliness (Zhao and Shen, 2011; Yue et al., 2014). Second, given that study quality may vary across studies and may affect the findings, we also tested its moderating effects on effect sizes.

In addition, publication bias was examined by using visual examination of funnel plots, fail-safe Ns (Rosenthal, 1979), and Egger's regression test analyses (Egger et al., 1997).

## Results

### Characteristics of Included Studies

In total, 51 studies published from 2008 to 2017 were included in the current review, with 96 effect sizes. A summary of the studies appears in Table 1.

**Table 1:** Summary of studies included in the meta-analyses.

**ID**	**Study**	**Publication type**	**Sample size (boys)**	**Number of effect sizes**	**Age range**	**Location**	**Study design**	**Measure (loneliness)**	**Measures (predictors)**	**Quality**
1	Liu et al., 2008	J	181(88)	4	10–16	Henan	Cross-sectional	CLS	SDI (gender); SRNQ (social support)	11
2	Rong, 2008	J	170(96)	1	Grades 4–6	Not reported	Cross-sectional	CLS	SDI (gender)	11
3	Xie, 2008	D	278(141)	2	Grades 7–9	Sichuan	Cross-sectional	LSA	SDI (gender); PMQ (both-parent migration)	9
4	Wang, 2008	J	730(n/a)	1	Grades 5–6	Sichuan	Cross-sectional	CLS	PMQ (both-parent migration)	7
5	Ren and Shen, 2008	J	262(141)	1	6–12	Zhejiang	Cross-sectional	CLS	SDI (gender)	7
6	Wu et al., 2010	J	437(n/a)	1	11–16	Guangdong	Cross-sectional	CLS	SSRS (social support)	9
7	Zhong et al., 2010	J	263(170)	1	Grades 7–9	Not reported	Cross-sectional	LSA	FAD (family functioning)	10
8	Sun et al., 2010	J	407(n/a)	1	Grades 4–6	Hunan	Cross-sectional	CLS	FQQ (friendship quality)	11
9	Qi and Jia, 2010	J	393(204)	2	11–17	Anhui	Cross-sectional	CLS	SDI (gender); PMQ (both-parent migration)	11
10	Wang, 2011	D	94(52)	3	Grades 4–6	Gansu	Cross-sectional	CLS	SDI (gender); PMQ (both-parent migration); SSRS (social support)	12
11	Zhao and Shen, 2011	J	207(123)	1	10–17	Henan	Cross-sectional	CLS	SDI (age); CALHS (Cognitive Appraisals for Left-home Hassles)	9
12	Zhang, 2011b	J	164(95)	2	Grades 3–5	Henan	Cross-sectional	CLS	PN (peer acceptance); ASS (parent-child relationship)	9
13	Zhang, 2011a	J	264(n/a)	4	Grades 3–9	Henan	Cross-sectional	CLS	SRNQ (social support); RSES (self-esteem)	9
14	Yang et al., 2013	J	205(101)	2	Grades 7–8	Chongqing	Cross-sectional	LSA	SDI (gender); NEO (extroversion)	11
15	Sun et al., 2013	J	288(159)	4	Grades 7–9	Hubei	Cross-sectional	UCLA	SDI (gender); PMQ (both-parent migration); FQQ (friendship quality); RSCA (resilience)	11
16	Wang, 2013	D	546(276)	1	6–14, Grades 1–6	Shandong	Cross-sectional	CLS	SDI (gender)	10
17	Dong and Zhang, 2013	J	713(371)	1	Grades 7–9	Yunnan	Cross-sectional	CLS	RSCA (resilience)	11
18	Zhao et al., 2013	J	209(120)	3	10–17	Henan	Cross-sectional	CLS	PN (peer acceptance); FACES (parent-child relationship); CBAS (beliefs about adversity)	11
19	Zhao, 2013	J	207(123)	3	10–17	Henan	Cross-sectional	CLS	SDI (gender);PMQ (both-parent migration); CRPBI (family functioning)	11
20	Duan, 2014a	J	184(111)	2	Grades 3–6	Xinjiang	Cross-sectional	CLS	SDI (gender);PMQ (both-parent migration)	11
21	Xu, 2014	D	306(137)	3	Grades 7–9	Anhui	Cross-sectional	LSA	SDI (gender); PHCSCS (self-esteem); FQQ (friendship quality)	9
22	Yuan et al., 2014	J	744(n/a)	1	Grades 3–6	Hebei	Cross-sectional	CLS	PMQ (both-parent migration)	10
23	Duan, 2014b	J	184(111)	2	Grades 3–6	Xinjiang	Cross-sectional	CLS	PHCSCS (self-esteem); SASC (social anxiety)	11
24	Sun, 2015	D	520(n/a)	2	Grades 3–7	Zhejiang, Jiangsu, Liaoning, & Anhui	Cross-sectional	CLS	PN (peer relationship); SPPC (self-esteem)	8
25	Yue and Lu, 2015	J	387(n/a)	2	Grades 4–6	Jiangsu & Guizhou	Cross-sectional	CLS	PMQ (both-parent migration)	11
26	Zhang and Hu, 2015	J	437(214)	1	Grades 5–6	Jiangxi	Cross-sectional	CLS	PHCSCS (self-esteem)	12
27	Zhao, 2015	J	366(192)	2	10–16	Guizhou	Cross-sectional	CLS	SSRS (social support); CSES (core self-evaluations)	11
28	Xiao and Zhang, 2015	J	437(214)	1	Grades 5–6	Jiangxi	Cross-sectional	CLS	SSRS (social support)	12
29	Fan et al., 2016	J	701(330)	3	Primary and Junior high school	Hunan	Cross-sectional	LSA	SDI (gender); PCQ (family functioning); CHS (trait hope)	11
30	Xu, 2016	J	628(316)	3	Grades 5–8	Henan & Anhui	Cross-sectional	CLS	SDI(gender)	12
31	Yang et al., 2016	J	985(488)	2	Grades 4–6	Hunan	Cross-sectional	CLS	APGAR (family functioning); PSSM (school sense of belonging)	12
32	Kong et al., 2016	J	474(206)	2	Grades 7–9	Shandong	Cross-sectional	UCLA	SDI (gender); SSRS (social support)	7
33	Liao et al., 2014	J	773(392)	3	9–16	Zhejiang	Cross-sectional	CLS	SDI (gender); SAS (social anxiety); SCSQ (coping styles)	11
34	Wang et al., 2011	J	250(n/a)	1	Grades 4–8	Not Reported	Cross-sectional	CLS	PMQ (both-parent migration)	10
35	Fan, 2011	J	545(269)	1	Grades 4–8	Hunan	Cross-sectional	CLS	PMQ (both-parent migration)	11
36	Liu et al., 2010	J	216(n/a)	1	Grades 4–6	Hubei	Cross-sectional	CLS	PHCSCS (self-esteem)	10
37	Ai and Hu, 2016	J	414(214)	2	10–13, primary school	Hunan & Sichuan	Cross-sectional	CLS	CDRS (resilience);SSRS (social support)	11
38	He, 2010	D	180(n/a)	1	Grades 8–9	Chongqing	Cross-sectional	UCLA	FAD (family functioning)	11
39	Fan et al., 2014	J	234/151(n/a)	3	Grades 4–7	Hunan	Longitudinal	LSA	FAS (family adversity); NEO (extroversion); RSES (self-esteem)	11
40	Duan and Zhang, 2014	J	435(238)	2	Grades 4–6	Gansu	Cross-sectional	CLS	CPANS (abuse and neglect);PHCSS (self-esteem)	10
41	Yue et al., 2014	J	311(149)	2	Grades 1–6	Hunan	Cross-sectional	LCLQ	SDI (age, gender); PCRQ (family functioning)	10
42	Liu et al., 2014	J	924(486)	1	Grades 1–5,7–10	Xinjiang	Cross-sectional	CLS	RSCA (resilience)	11
43	Liu, 2014	J	301(156)	2	Grades 3–6	Hebei	Cross-sectional	CLS	SDI(gender); PHCSCS (self-esteem)	12
44	Xu, 2008	D	114 (n/a)	2	Grades 3–5	Hubei	Cross-sectional	CLS	SDI (gender); CEQ (teacher-student relationship, peer relationship)	8
45	Zhao et al., 2008	J	218(n/a)	1	Grades 4–8, 10–17	Henan	Cross-sectional	CLS	PMQ (both-parent migration)	11
46	Zhao et al., 2015	J	241(140)	2	Grades 4–8, 10–17	Henan	Cross-sectional	CLS	FACES (parent-child relationship); NRI (friendship quality)	14
47	Zhang and Hu, 2015	CP	178(109)	1	Grades 4–6	Hubei	Cross-sectional	CLS	SDI (gender)	11
48	Su et al., 2013	J	501(249)	1	Grades 5–8	Guangxi	Cross-sectional	CLS	PMQ (both-parent migration)	12
49	Song et al., 2017	J	427(275)	3	Grades 7–8	Hubei	Cross-sectional	CLS	PN (peer relationship); FACES (parent-child relationship); RSES (self-esteem)	13
50	Yu, 2017	J	305(157)	2	Grades 4–6	Guangdong	Cross-sectional	CLS	SCS (social support)	12
51	Ren et al., 2017	J	416(211)	1	Junior and senior school	Hunan	Cross-sectional	UCLA-8	SAS (social anxiety)	12

*Measures: APGAR, Family APGAR Index; CALHS, Cognitive Appraisals for Left-home Hassles Scale; CBAS, Chinese Beliefs about Adversity Scale; CDRS, Connor–Davidson Resilience Scale; CEQ, Class Environment Questionnaire; CHS, Children's Hope Scale; CLS, Children's Loneliness and Social Dissatisfaction Scale; CRPBI, Child Report of Parent Behavior Inventory; CSES, Core Self-Evaluations Scale; FACES, Family Adaptability and Cohesion Evaluation Scales; FAD, Family Assessment Device; FAS, Family Adversity Scale; FQQ, Friendship Quality Questionnaire; LCLQ, Left-behind children's Loneliness Questionnaire; LSA, Loneliness Scale of Adolescent; NEO, NEO Personality Inventory; NRI, Network of Relationships Inventory; PCQ, Parental Care Questionnaire; PCRQ, Parent-Child Relationship Questionnaire; PHCSCS, Piers-Harris Children's Self-Concept Scale; PMQ, Parental Migration Questionnaire; PN, Peer Nomination; PSSM, Psychological Sense of School Membership Scale; RSCA, Resilience Scale for Chinese Adolescents; RSES, Rosenberg's Self-Esteem Scale; SAS, Social Anxiety Scale; SCS, School Climate Scale; SCSQ, Simplified Coping Style Questionnaire; SDI, Standard demographic information; SM, Sociometric Methods; ASS, Attachment Security Scale; SPPC, Harter's Self-perception Profile for Children; SRNQ, Social Relations Network Questionnaire; SSRS, Social Support Rate Scale; UCLA, UCLA Loneliness Scale; Publication type: D, Master's thesis; J, journal article; CP, Conference Proceedings*.

**Figure 1:**
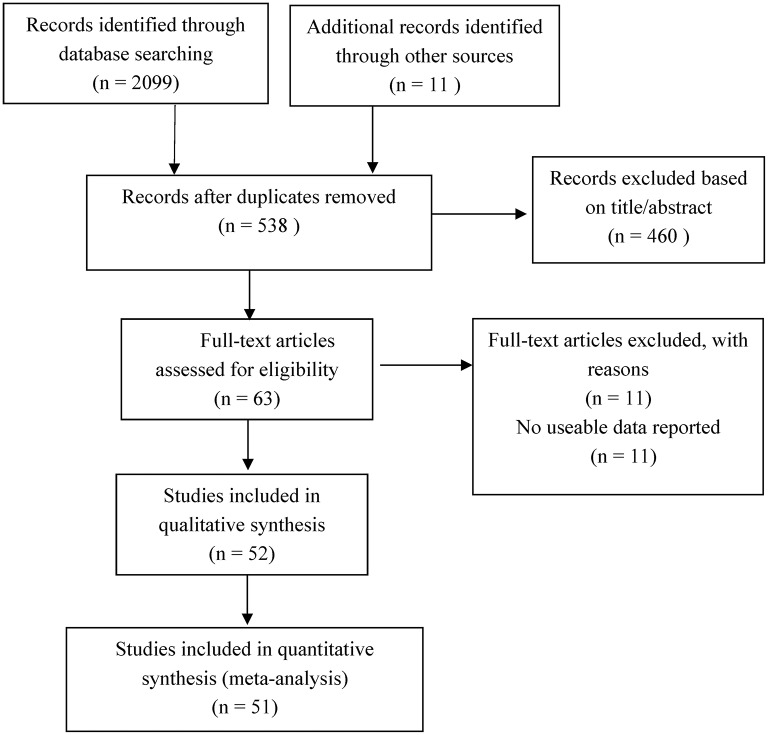
PRISMA flow diagram.

Among the 51 studies, except for one longitudinal design (Fan et al., 2014), the other studies were all cross-sectional. Study sample sizes ranged from 94 to 985. As for type of publication, 43 were journal articles, 7 were master's theses, and one was a conference article. With respect to measures of children's loneliness, the most frequently used scale (40 studies, 78.34% of studies eligible in this meta-analysis) was the Chinese version of Children's Loneliness and Social Dissatisfaction Scale (Asher et al., 1984). Other measures included the Loneliness Scale of Adolescents (Zou, 2003), revised version of UCLA Loneliness Scale (Russell, 1996), and Left-behind children's Loneliness Questionnaire (Yue et al., 2014). Figure 1 shows the complete selection process.

### The Outcome of Meta-Analysis

The factors associated with loneliness in left-behind children appear in Table 2, illustrating the number of studies, effect size, and 95% confidence intervals. Table 2 also provides information on heterogeneity and publication bias. The forest plots diagrams for each meta-analysis are presented in Appendix C.

**Table 2:** Meta-analyses of individual and contextual factors for loneliness among left-behind children.

	**Effect sizes**					**Heterogeneity**				**Publication bias**	
	***k***	***r***	**95% CI**	**Z**	***p***	***Q***	***p***	***I^**2**^***	**τ^2^**	**Fail-safe N**	**Eggers test two-tailed *p***
**INDIVIDUAL FACTORS**											
Gender(boy)	20	0.07	[0.03, 0.11]	3.13	0.002	55.63	0.000	68.84	0.01	106	0.50
Older age	2	−0.14	[−0.22, −0.05]	−3.14	0.002	0.175	0.68	0.00	0.000	n/a	n/a
Self-esteem	9	−0.42	[−0.51, −0.33]	−8.30	0.000	71.68	0.000	88.84	0.02	1,409	0.64
Resilience	4	−0.37	[−0.48, −0.24]	−5.54	0.000	30.84	0.000	90.27	0.02	295	0.09
Extroversion	2	−0.40	[−0.48, −0.32]	−8.79	0.000	0.25	0.62	0.00	0.000	n/a	n/a
Social anxiety	3	0.49	[0.43, 0.55]	13.49	0.000	3.72	0.16	46.21	0.002	279	0.72
**CONTEXTUAL FACTORS**											
Family functioning	6	−0.27	[−0.33, −0.21]	−8.33	0.000	11.57	0.04	56.80	0.003	258	0.43
Parent-child relationship	4	−0.31	[−0.36, −0.25]	−10.19	0.000	0.33	0.95	0.00	0.000	101	0.77
Both-parent migration	19	0.004	[−0.02, 0.03]	0.33	0.74	16.21	0.58	0.00	0.000	0	0.73
Mother-only migration	5	−0.02	[−0.11,0.14]	0.25	0.81	18.85	0.001	78.78	0.016	0	0.97
Peer relationship	9	−0.45	[−0.56, −0.31]	−5.81	0.000	137.63	0.000	94.19	0.06	1,337	0.88
Social support	8	−0.40	[−0.50, −0.28]	−6.15	0.000	91.87	0.000	92.38	0.03	1,072	0.28

*k, number of studies; 95% CI, 95% confidence intervals around the effect size*.

#### Individual Factors

Meta-analyses on the associations between several individual factors (demographic variables, self-esteem, resilience, personality traits, and social anxiety) and loneliness among left-behind children were conducted. First, we tested the relationships between demographic variables and loneliness. We found that some demographic variables (i.e., gender, age) were associated with loneliness among left-behind children. Gender showed a minimal effect size (*k* = 20, *r* = 0.07; 95% CI: 0.03–0.11, *p* < 0.01). Specifically, boys were more likely to be lonely than girls. Moreover, there was moderate heterogeneity in effect sizes between studies (*Q* = 55.63, *p* < 0.0001, *I*^2^ = 68.84%, τ^2^ = 0.01). Subgroup analysis showed that effect sizes did not vary by age groups (*Q*_*b*_ = 3.09, *p*_*b*_ = 0.21). Meta-regression showed that the study quality rating score could not account for heterogeneity (slope = 0.01, *p* = 0.63). With respect to age, older left-behind children experienced less loneliness with a small effect size (*k* = 2, *r* = −0.14; 95% CI: −0.22 to −0.05, *p* < 0.01). The overall effect size was not heterogeneous (*Q* = 0.18, *p* = 0.68, *I*^2^ = 0, τ^2^ < 0.0001).

Second, we conducted meta-analysis to examine the associations between self-esteem and loneliness. Results showed that higher self-esteem was associated with less loneliness with a medium effect size (*k* = 9, *r* = −0.42; 95% CI: −0.51 to −0.33, *p* < 0.0001). Moderate heterogeneity was found in the studies (Q = 71.681, *p* < 0.0001, *I*^2^ = 88.84%, τ^2^ = 0.02). Subgroup analysis showed that there was no significant difference in effect sizes across different age groups (*Q*_b_ = 0.28, *p*_*b*_ = 0.59). Meta-regression showed that the study quality rating score could not account for the heterogeneity (slope < 0.01, *p* = 0.84).

Third, children with higher levels of resilience experienced less loneliness with a medium effect size (*k* = 4, *r* = – 0.37; 95% CI: −0.48 to −0.24, *p* < 0.0001). There was high heterogeneity in effect sizes across studies (*Q* = 30.84, *p* < 0.001, *I*^2^ = 90.27%, τ^2^ = 0.02).

Fourth, extroverted children experienced less loneliness with a medium effect size (*k* = 2, *r* = −0.40; 95% CI: −0.48 to 0.32, *p* < 0.0001). No heterogeneity was found across studies (*Q* = 0.25, *p* = 0.62, *I*^2^ = 0, τ^2^ < 0.0001).

Finally, children with higher levels of social anxiety experienced more loneliness with a medium effect size (*k* = 3, *r* = 0.49; 95% CI: 0.43 to 0.55, *p* < 0.0001). Moderate heterogeneity was found across studies (*Q* = 3.72, *p* = 0.16, *I*^2^ = 46.21%, τ^2^ <0.01).

#### Contextual Factors

We also examined the predicting effects of several contextual factors, including family environment (e.g., family functioning, parent-child relationship, parental migration status), school environment (e.g., peer relationship), and social support on loneliness. As for family environment, positive family functioning was found to correlate with low levels of loneliness among left-behind children with a small mean effect size (*k* = 6, *r* = −0.27; 95% CI: −0.33 to −0.21, *p* < 0.0001). Moderate heterogeneity (*Q* = 11.57, *p* = 0.04, *I*^2^ = 56.80%, τ^2^ < 0.01) occurred. Meanwhile, positive parent-child relationship was associated with lower levels of loneliness with a medium mean effect size (*k* = 4, *r* = −0.31; 95% CI: −0.36 to −0.25, *p* < 0.0001). No heterogeneity was found across studies (*Q* = 0.33, *p* = 0.95, *I*^2^ = 0, τ^2^ < 0.0001). Finally, results showed that no differences in average correlation of loneliness in the two parental migration status (both-parent migration vs. one-parent migration) (*k* = 19, *r* = 0.004; 95% CI: −0.02 to 0.03, *p* = 0.74). No heterogeneity in effect size estimates (*Q* = 16.21, *p* = 0.58, *I*^2^ = 0, τ^2^ < 0.01) occurred. In addition, for mother-only migration (vs. father-only migration), there is no clear evidence of an association with higher levels of loneliness (*k* = 5, *r* = 0.02; 95% CI: −0.11 to 0.14, *p* = 0.81), and high heterogeneity (*Q* = 18.85, *p* < 0.01, *I*^2^ = 78.78%, τ^2^ = 0.02) occurred.

With respect to school environment, positive peer relationship was related to less loneliness with a medium mean effect size (*k* = 9, *r* = −0.45; 95% CI: −0.56 to −0.31, *p* < 0.0001). High heterogeneity occurred (*Q* = 137.63, *p* < 0.0001, *I*^2^ = 94.19%, τ^2^ = 0.06). Subgroup analysis showed no evidence that effect size differed by age group (*Q*_*b*_ = 0.36, *p*_*b*_ = 0.55). Meta-regression revealed that the study quality rating score could account for the heterogeneity (slope = 0.07, *p* < 0.0001).

In addition, social support was related to children' loneliness with a medium mean effect size (*k* = 8, *r* = −0.40; 95% CI: −0.50 to −0.28, *p* < 0.0001). High heterogeneity was found in the studies (*Q* = 91.87, *p* < 0.000, *I*^2^ = 92.38%, τ^2^ = 0.03). Subgroup analysis showed no evidence that effect size differed by age group (*Q*_*b*_ = 1.07, *p*_*b*_ = 0.30). Meta-regression showed that the study quality rating score could account for the heterogeneity (slope = 0.07, *p* < 0.0001).

#### Publication Bias

No publication bias was found in the meta-analysis for all outcomes according to the Eggers test or Rosenthal's failsafe number (see Table 2). In addition, the funnel plot of each predictor was generally symmetrical (see Appendix D). In short, the risk for publication bias in this meta-analysis can be considered low.

## Discussion

Grounded in an ecological systems perspective, this meta-analysis review examined what factors are associated with loneliness among left-behind children in rural China. Specifically, some key individual factors and contextual factors for loneliness among these children were identified across 51 studies published between 2008 and 2017.

With respect to individual factors, demographic factors (i.e., gender, age) and intrapersonal psychological characteristics (i.e., self-esteem, resilience, extroversion, and social anxiety), were explored in this review. We found a minimal but significant gender differences in the feeling of loneliness based on 20 studies: boys experienced higher levels of loneliness as compared to girls. One possible explanation for this finding is that, suppression may benefit interpersonal adaptation in Chinese culture (Butler et al., 2007), and Chinese boys are likely to be encouraged to suppress rather to express negative emotions (Sun et al., 2010). According to gender schema theory, children may develop a schema to fit gender roles in a unique cultural environment (Martin and Halverson, 1981): “I am a boy, so I can't cry and I am tough.” What's more, suppression may increase memory for negative emotions during tense social interactions (Richards et al., 2003). Another reason may be that boys are more likely to show social and school adjustment problems than girls, which may relate to loneliness (Chen et al., 2001). Researchers, however, remain split on gender difference in the severity of loneliness in children and adolescents (Koenig and Abrams, 1999; Weeks and Asher, 2012). Therefore, researcher should further explore gender differences in loneliness in different socio-cultural contexts.

In terms of age, the findings show a small but significant effect on loneliness. Consistent with previous studies (Harris et al., 2013; Ladd and Ettekal, 2013), normative (mean-level, or average) changes in loneliness tend to decline during childhood and adolescence. The age differences in loneliness may result from age differences in children's social and cognitive competence (Laursen and Hartl, 2013). Older children may be able to reappraise change in family structure caused by parental migration and regulate the negative emotional impact. Evidence from a qualitative study indicated that the ability to construct positive meaning from parental migration increased with age (Fu and Law, 2018). Therefore, older age may be a protective factor for loneliness among left-behind children.

In addition, intrapersonal psychological characteristics are also important predictors of loneliness among left-behind children. First, the strongest effect on loneliness were related to self-esteem with a medium effect size in the current review. This finding, to some extent, supports the cognitive discrepancy model (Peplau and Perlman, 1982), which posits that children with low levels of self-esteem are likely to engage in certain irrational cognition and behaviors and will not establish and maintain satisfactory social relationships, which contribute to loneliness. Also, according to the social exclusion theory (Baumeister and Tice, 1990) and sociometer theory (Leary et al., 1995), self-esteem is an interpersonal monitor, which reflects the individual historical experience of social exclusion and inclusion, and there may be a reciprocal relationship between self-esteem and loneliness. People with low self-esteem are more likely to feel real and imagined threats related inclusion in social context, providing an explanation for why they are more likely to be lonely; in turn, lonely people may blame themselves for being isolated and thus damage the self-esteem system (Leary, 1990; Leary et al., 1995). Indeed, the emerging empirical literature have attested to this reciprocal relationship between them (e.g., Vanhalst et al., 2013; Du et al., 2019).

Second, we found that extroverted left-behind children appeared to be less lonely than introverts perhaps because extroverts have large social networks and high-quality social relationships (Levin and Stokes, 1986; Lopes et al., 2003). Third, we also found that resilience is a protective factor for children's loneliness. Resilience has been found to act as a buffer between adversity (e.g., absence of parental care, poor social support) and loneliness among left-behind children (Liu et al., 2014; Ai and Hu, 2016). Specifically, developing resilience can help children build self-confidence and optimism against the risk of loneliness (Fergus and Zimmerman, 2005; Ai and Hu, 2016).

Finally, the results of this review coincide with a prior meta-analysis (Mahon et al., 2006) in which high social anxiety was found to be a risk factor for children's loneliness. Higher levels of social anxiety have been linked with lower levels of peer relations and friendships (Greca and Lopez, 1998), and negative peer consequences related to social anxiety may increase feeling of loneliness among left-behind children. We cannot, however, elucidate the direction of the association because of most studies in this meta-analysis are cross-sectional. A reverse interpretation (that is, loneliness leads to social anxiety) and a bidirectional relationship may exist. Further longitudinal studies are needed to explore the relationships between social anxiety and loneliness among left-behind children.

As far as contextual factors are concerned, we found some significant protective factors within family and school contexts that had small to medium effect sizes through this meta-analysis. Within family context, family functioning is a key predictor of loneliness in left-behind children. Consistent with previous studies, children who perceived positive family functioning always reported lower levels of loneliness (Sturge-Apple et al., 2010; Sharabi et al., 2012). Conversely, family dysfunction (e.g., improper parenting style) can increase the likelihood of loneliness in children through insecure attachment (Rotenberg, 1999). In addition, positive parent-child relationship plays a protective role for loneliness among left-behind children. Moreover, one may suspect that both-parent migration is a risk factor and children in that situation may experience higher levels of loneliness than children with one-parent migration; however, no evidence was found for an association between parental migration status and loneliness in this meta-analysis. Further efforts are therefore required to explore the reason why the parental migration status was not related to children's loneliness.

In addition to family, school is also an important ecological context for children' loneliness. Consistent existing studies (Asher and Paquette, 2003), our results indicated that peer relationship was significantly associated with children's loneliness. According to the theory of social loneliness (Weiss, 1973), the key marker of feeling loneliness is a lack of close and satisfying relationships. As such, peer relationship is a well-established predictor of loneliness among left-behind children.

Finally, consistent with the results of a previous meta-analysis (Masi et al., 2011), multiple sources of social support play an important role in buffering the risk of children's loneliness. Higher levels of support across sources (e.g., parents, peers, teachers) may provide children with a strong sense of belonging and companionship and then alleviate the feeling of loneliness (Cavanaugh and Buehler, 2016); therefore, enhancing social support may be an important intervention strategy to reduce loneliness among left-behind children.

### Moderator Analysis of Factors Associated With Loneliness

Moderator analysis for factors with moderate to high heterogeneity (resilience, social anxiety, and family functioning were excluded because of the small number of studies) showed that the study quality was a significant moderator of the relationships between peer relationship, social support, and loneliness. Specifically, higher-quality studies showed smaller effect sizes than lower-quality studies. This finding means that the methodological quality of studies should be considered when understanding these effect sizes.

### Limitations and Implications

Several limitations should be noted in this review. First, most of the eligible studies involved cross-sectional data, limiting our capability to evaluate the appropriateness of causal inferences to evaluate the appropriateness of causal inferences. Future meta-analysis may be needed when more longitudinal studies emerge. Second, given that there are not many studies focusing on the predictors of loneliness, in the analyses we only examined a relatively small number of studies for each factor that was included. Third, we were not able to control for other factors when examining the effects of each factor. Further meta-analysis is needed to address these issues when more studies are filled into this field. Fourthly, meta-analysis showed some factors in high heterogeneity, suggesting that a potential moderator can explain the differences in effect sizes. Unfortunately, because of the small number of studies, we only tested two moderators, and their moderating effects were mostly not significant. Finally, based on the location characteristics of included studies (see Table 1) and previous research (e.g., He, 2008), some parents of children may work abroad, not in China, especially in the coastal areas of southeastern China (e.g., Zhejiang, Guangdong). The studies included in this review, unfortunately, didn't clearly report relevant information. Therefore, it remains unclear whether the two groups differ in these associations. Further research should focus on this issue by adopting a comparative approach.

Despite the limitations, the present study has several important implications for future research and practice focusing on the reduction of loneliness and the promotion of well-being among left-behind children. First, a developmental and ecological systems perspective can be used in examining the effects of individual and contextual variables on the development of loneliness in left-behind children in future research. Second, parenting training can be provided to help parents improve the frequency and quality of communication with children and enhance family warmth and emotional connections among family members. In addition, school personnel are encouraged to create a positive school climate (e.g., support, caring, respect, equality, positive expectations) by providing effective left-behind children development programs. For example, out-of-school time programs emphasizing on improving peer relationships, skills of seeking social support, and other intrapersonal strengths (e.g., resilience and self-esteem) can be offered to left-behind children to reduce their loneliness and promote their positive development. Moreover, social security and protection systems associated with the development of left-behind children should be established through the collaboration of families, schools, and communities and can, for example, focus on increasing investment in rural communities and education, caring for migration families, and improving caregivers' parenting skills by offering parenting lessons.

## Conclusion

Framed within the ecological systems model, this meta-analytic review identified small to medium effects for a variety of factors that predict left-behind children's loneliness. Overall, this review identified one individual risk factor (i.e., social anxiety) and several individual (i.e., older age, self-esteem, resilience, extroversion) and contextual (i.e., family functioning, parent-child relationship, peer relationship, and social support) protective factors for loneliness among left-behind children. Despite the methodological limitations of the studies included in this meta-analysis, these findings are important for understanding how to reduce loneliness and promote well-being among left-behind children.

## Author Contributions

XC, XL, and DL collaboratively designed this meta-analysis. XC and XL conducted the literature search and coded studies. XC and DL did data analysis and wrote the first draft of the manuscript. HD provided valuable ideas and revised the manuscript. SS helped revise the manuscript.

### Conflict of Interest Statement

The authors declare that the research was conducted in the absence of any commercial or financial relationships that could be construed as a potential conflict of interest.
