# Supplementary material for: What Really Matters for Loneliness Among Left-Behind Children in Rural China: A Meta-Analytic Review
Source: Front Psychol. 2019 Apr 18;10:774. doi: 10.3389/fpsyg.2019.00774 (PMC6482215; doi:10.3389/fpsyg.2019.00774)
Supplement: Supplementary file 1 [file Data_Sheet_1.PDF]

Supporting Information

Appendix A. Search strategy

Chinese electronic databases: CNKI, China Dissertation Database

#1 liushou(留守)*

#2 ertong(儿童)*or qingshaonian(青少年)*

#3 gudu(孤独)*or yuce (预测)* or fengxian(风险)* or baohu (保护)*

Searching: 1 and 2 and 3

English electronic databases: PubMed, Web of Science, PsycInfo and ProQuest

#1 left-behind*

#2 children* or adolescent*

#3 loneliness* or predictor* or risk * or protective*

4 China or Chinese

Searching: 1 and 2 and 3 and 4

Literature search – Google

Chinese: (Liushou ertong(留守儿童) or Liushou qingshaonian(留守青少年)) and (gudu(孤独))

English: (LBC or left-behind adolescent) and (loneliness)

Appendix B. The modified quality index

Reporting

1. Is the hypothesis/objective of the study clearly described?

2. Are the main outcomes to be measured clearly described in the Introduction or Methods section?

3. Are the characteristics of the patients included in the study clearly described?

4. Are the main findings of the study clearly described?

5. Does the study provide estimates of the random variability in the data for the main outcomes?

6. Have actual probability values been reported for the main outcomes except where the probability value is less than 0.001?

7. Is the response rate clearly described?

External validity

8. Were the subjects asked to participate in the study representative of the entire population from which they were recruited?

9. Were subjects who were prepared to participate representative of the entire population from which they were recruited?

Internal validity (bias and confounding)

10. If any of the results of the study were based on ‘‘data dredging,’’ was this made clear?

11. Were the statistical tests used to assess the main outcomes appropriate?

12. Were the main outcome measures used valid and reliable?

13. Was there adequate adjustment for confounding in the analyses from which the main results were drawn?

Power

14. Did the study provide a sample size or power calculation to detect important effects where the probability value for a difference being due to chance is less than 0.05?

References

Ferro, M. A., & Speechley, K. N. (2009). Depressive symptoms among mothers of children with epilepsy: A review of prevalence, associated factors, and impact on children. *Epilepsia, 50*, 2344-2354.

Appendix C: Overall Forest plots for data from meta-analysis

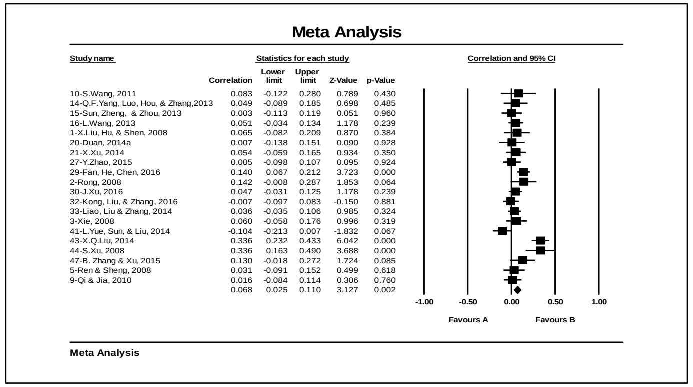

Figure 1. Gender(boy)

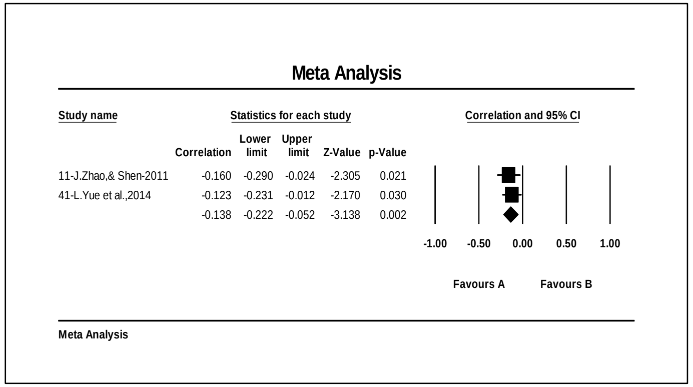

Figure 2. Older Age

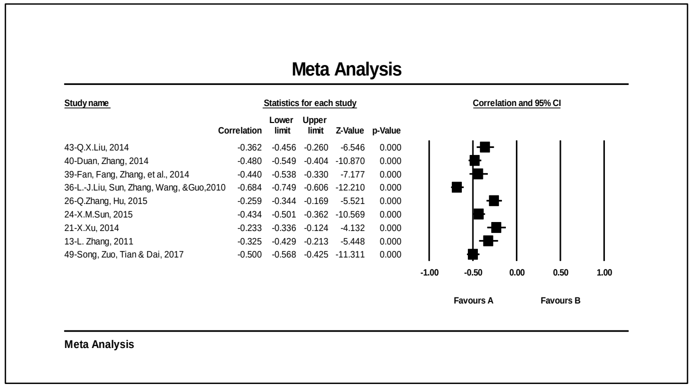

Figure 3. Self-Esteem

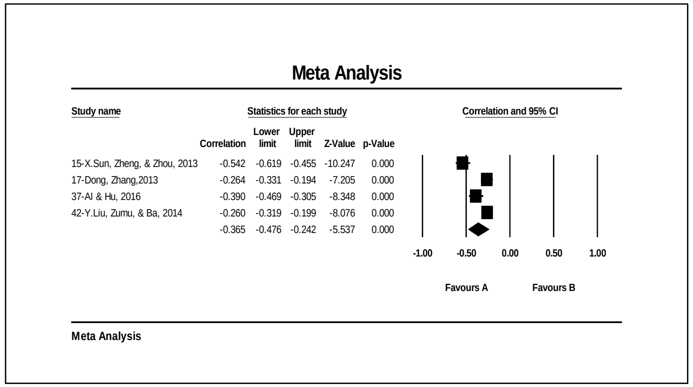

Figure 4. Resilience

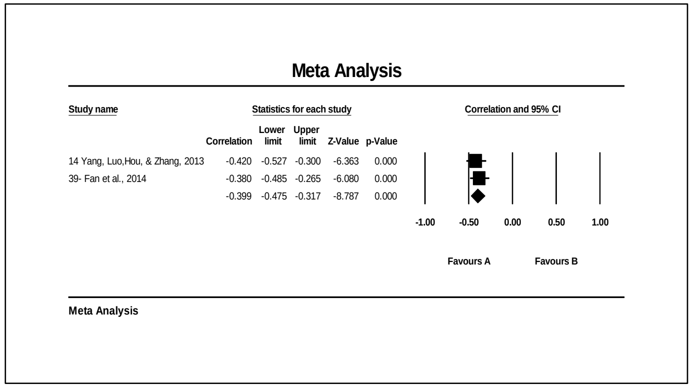

Figure 5. Extroversion

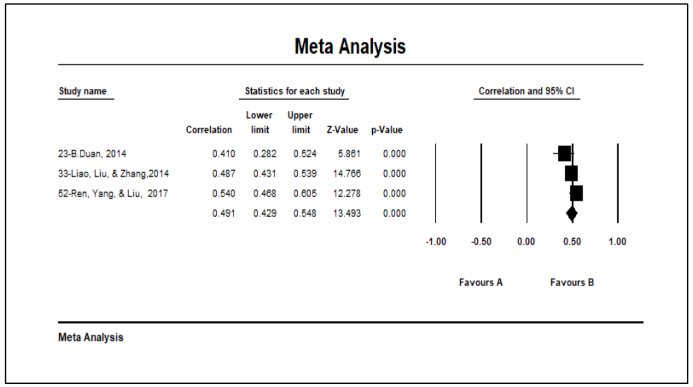

Figure 6. Social Anxiety

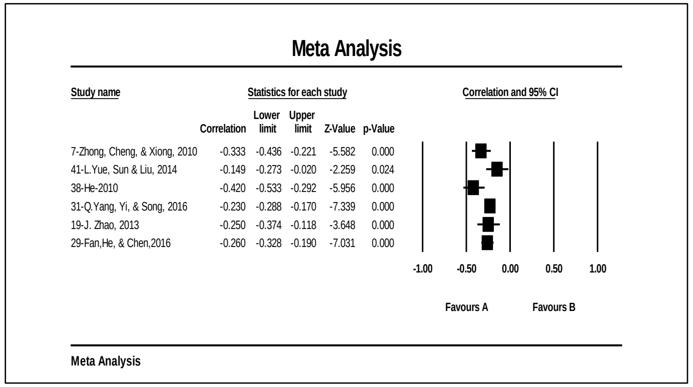

Figure 7. Family Functioning

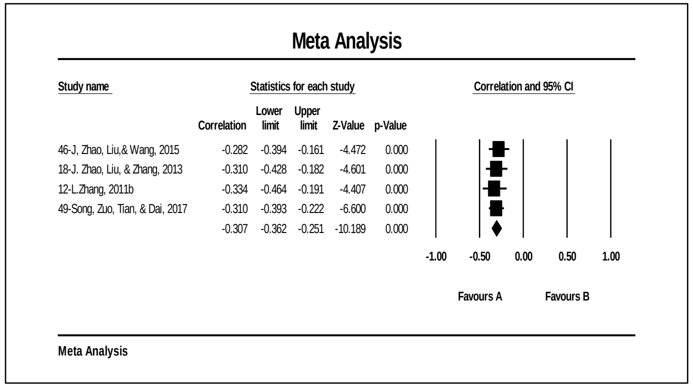

Figure 8. Parent-Child Relationship

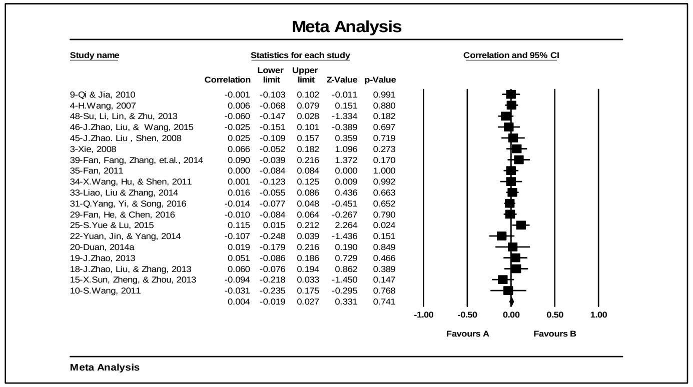

Figure 9. Both Parent-Migration (vs. one parent migration)

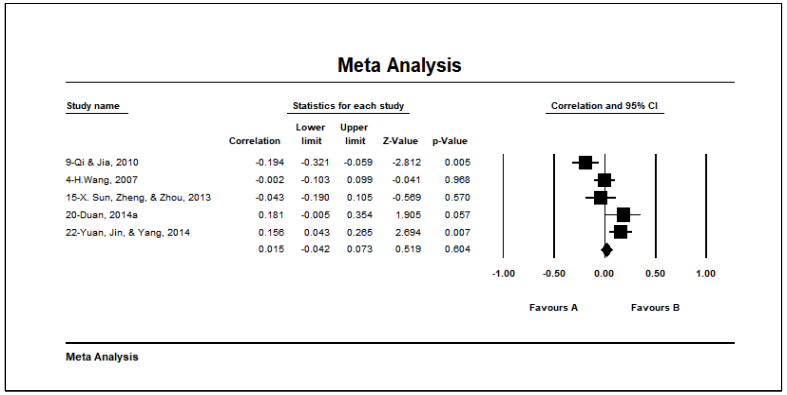

Figure 10. Mother-only migration (vs. Father-only migration)

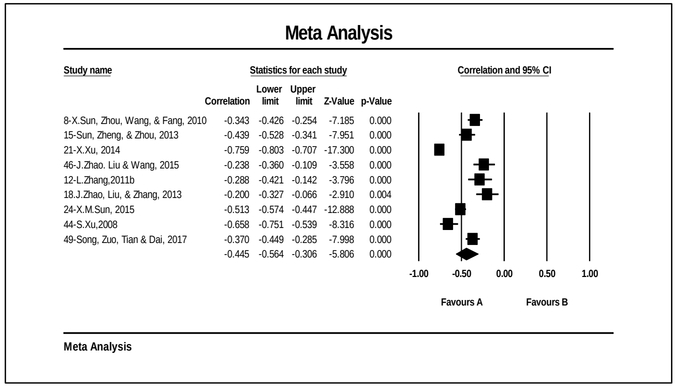

Figure 11. Peer Relationships

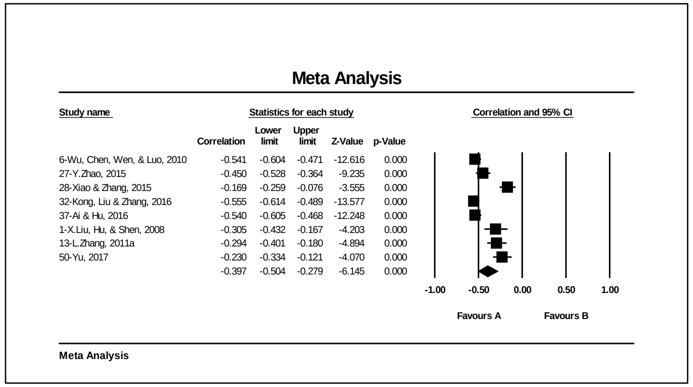

Figure 12. Social Support

Appendix D: Publication bias funnel plots

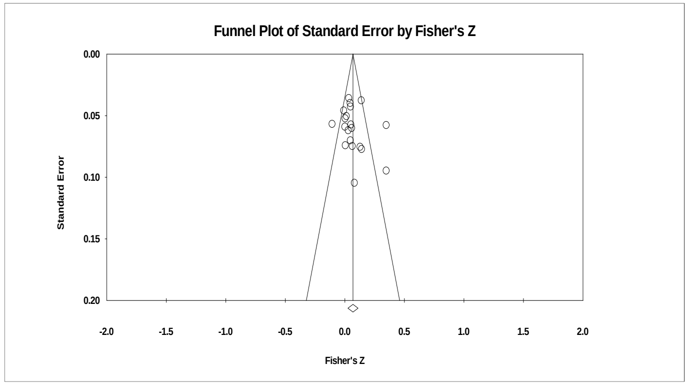

Figure 1. Gender (boy)

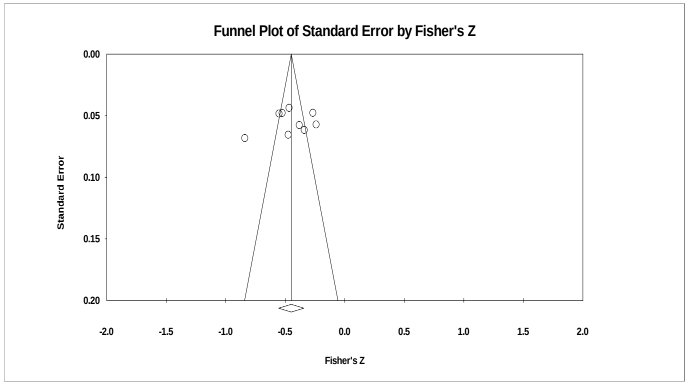

Figure 2. Self-Esteem

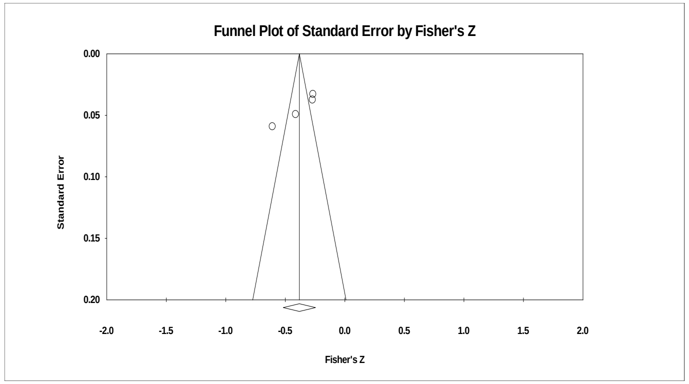

Figure 3. Resilience

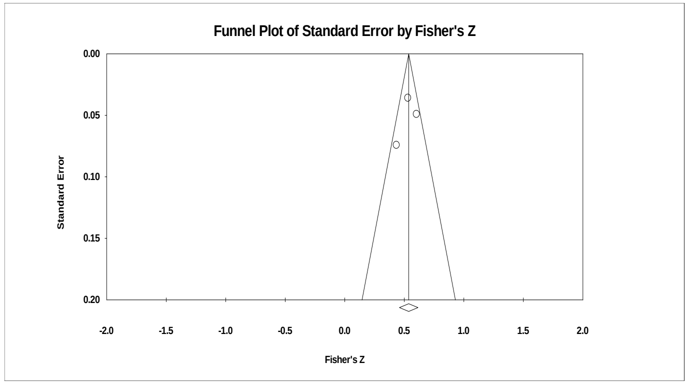

Figure 4. Social Anxiety

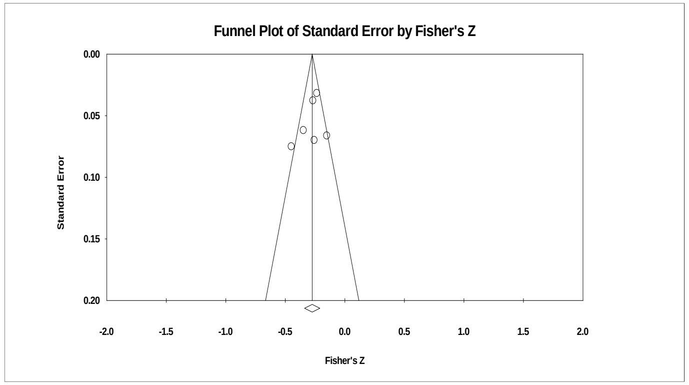

Figure 5. Family Functioning

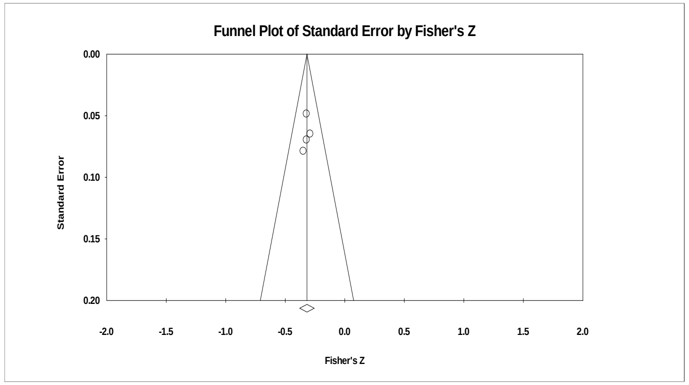

Figure 6. Parent-Child Relationship

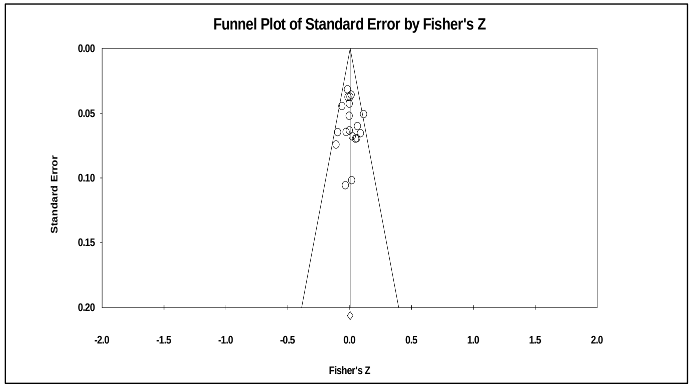

Figure 7. Both-Parent Migration (vs. one parent migration)

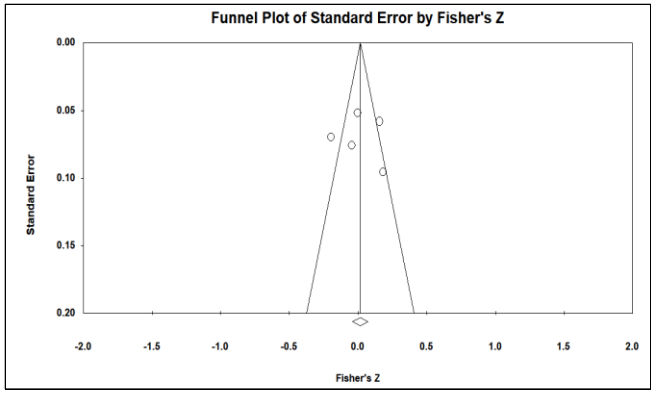

Figure 8. Mother-only migration (vs. Father-only migration)

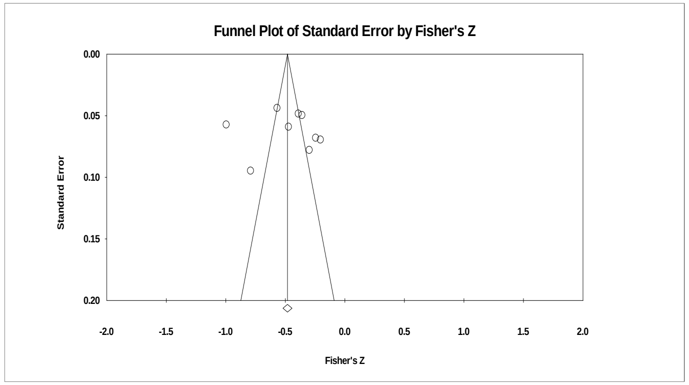

Figure 9. Peer Relationships

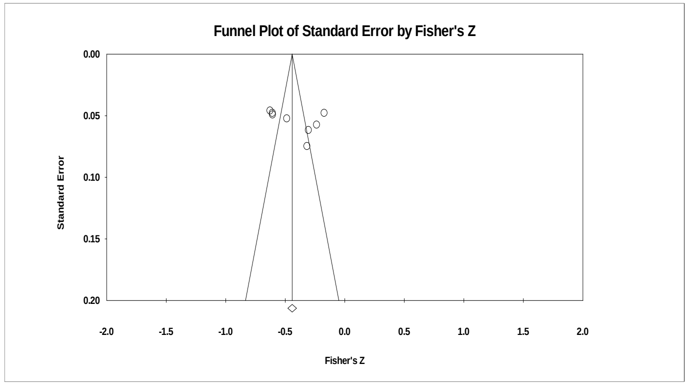

Figure 10. Social Support
